# New instrument for effective detection of a history of COPD exacerbations, including usually unreported events

**DOI:** 10.3389/fmed.2025.1630338

**Published:** 2025-09-19

**Authors:** Jaromir Zatloukal, Eva Volakova, Jana Kovacikova, Martina Kulirova, Miroslav Maruscak, Blanka Hytychova, Vladimir Koblizek

**Affiliations:** ^1^Department of Respiratory Medicine, University Hospital Olomouc, Olomouc, Czechia; ^2^Faculty of Medicine and Dentistry, Palacky University, Olomouc, Czechia; ^3^Department of Pneumology, University Hospital Hradec Kralove, Hradec Kralove, Czechia; ^4^Faculty of Medicine in Hradec Kralove, Charles University, Hradec Kralove, Czechia; ^5^AstraZeneca Czech Republic, Prague, Czechia

**Keywords:** obstructive lung disease, structured checklist, questionnaire, flare-up, disease history, dyspnea, cough

## Abstract

**Background:**

COPD exacerbations are important events for disease management. The incidence of exacerbations impacts prognosis, guides treatment, and predicts future exacerbations. Despite their importance, exacerbations are often underdiagnosed and underreported. The aim of our study was to test and evaluate the effectiveness of a structured checklist for detecting past exacerbations that we developed and that would be suitable for routine clinical practice.

**Methods:**

350 patients with COPD and FEV_1_ < 80% of the predicted value were enrolled in 35 centers. Each patient completed a structured checklist and underwent an interview with the physician. The number of exacerbations, their symptoms and duration, and the treatments were evaluated. Clinical data on exacerbations in the previous 12 months were retrieved from the patient’s medical records and analyzed retrospectively. The data obtained using the structured checklist were compared with the data from the interviews and medical records.

**Results:**

Compared to the patient-physician interview, the structured checklist detected more exacerbations since the previous visit (*p* = 0.025). The difference was significant also for severe exacerbations (*p* = 0.003). In patients reporting only one event, the structured checklist was more sensitive in detecting mild events than the interview (*p* < 0.001). The structured checklist detected mild exacerbations in 10 patients in whom the interview detected none. Compared to the number of exacerbations in the medical records, the structured checklist detected more than twice as many events. The mean duration of an exacerbation was 9.7 days, and the most prominent symptoms were dyspnea and productive cough.

**Conclusion:**

Proposed structured checklist improved the detection of past exacerbations, including usually unreported events. Moreover, the structured checklist allows the severity and other clinical characteristics of past exacerbations to be specified and used to direct further COPD therapy.

## Introduction

Exacerbations of COPD are acute events characterized by increased respiratory symptoms (1, 2). Their impact and consequences are substantial for the patient, the disease management, and the healthcare system. Exacerbations negatively impact the patient’s health status, often for a prolonged time, and contribute to disease progression as they accelerate the decline of lung functions. Patients with frequent exacerbations, defined as two or more exacerbations per year, have worse health status and morbidity than patients with less frequent exacerbations. The frequency and severity of exacerbations are associated with the risk of hospitalizations, all-cause mortality, and COPD-related mortality (1–5). Exacerbations account for the most significant proportion of healthcare costs of COPD (6).

The number of exacerbations in the prior year is the strongest predictor of a patient’s future exacerbation frequency (1, 7). The history of these acute events is essential for optimizing the treatment. For example, the administration of inhaled corticosteroids is indicated in patients with an increased number of eosinophils in the peripheral blood and a history of exacerbations. Similarly, exacerbations are required to indicate treatment with biologics.

Despite their importance, about 50 to 67% of COPD exacerbations are unreported to physicians (8, 9). Although often shorter in duration, unreported events also have a significant impact on health status (1).

The history of exacerbations is usually screened during a physician-patient interview and/or from the medical records. The use of other instruments is limited in routine clinical practice. Established questionnaires and scales, such as The COPD Assessment Test (CAT), Clinical COPD Questionnaire score (CCQ), Modified Medical Research Council Dyspnea Scale (mMRC), or St. George’s Respiratory Questionnaire (SGRQ), are focused more on symptoms, quality of life, or early detection of exacerbations (10, 11), but are not designed to screen exacerbation history.

Exacerbations can also be detected and monitored using diaries completed by the patient on a daily basis (12). However, patients using paper-based data recording in studies of respiratory diseases often falsify data (13–15). Patients are probably more compliant with electronic records. In one randomized trial of electronic symptom recording vs. monitored paper-based recording, actual compliance was 94% in the electronic group, whereas compliance with paper-based recording was 73% (13, 16). Smartphone-based diary of COPD symptoms enables near-complete identification of exacerbations at onset (13), and this tool was employed in the FLAME (17) and SPARK (18) studies. The EXAcerbations of Chronic pulmonary disease Tool (EXACT) is another standard, validated instrument for quantifying the frequency, severity, and duration of exacerbations of COPD in clinical trials. Nevertheless, smartphone-based e-diaries and EXACT are still used in research rather than clinical practice (19, 20).

Our study aimed to test and evaluate the effectiveness of a structured checklist that we developed to detect past exacerbations and that would be suitable for routine clinical practice, by comparing it with patient-physician interviews and medical records.

## Materials and methods

### Research

The study was conducted during May and June 2023 and involved 35 outpatient pneumology centers. There are 14 administrative regions in the Czech Republic and 2–3 centers were selected from each region to cover the entire Czech Republic. Each center enrolled 10 consecutive COPD patients. Inclusion criteria were COPD with forced expiratory volume in 1 s (FEV_1_) < 80% of predicted value, duration of COPD at least 2 years, and follow-up at the respective center with complete medical records for at least 2 years. No other eligibility criteria were defined.

During the study visit, a routine patient-physician interview took place in which the investigator asked about the number of exacerbations the patient had experienced since the last visit. After a standard examination, each patient completed the structured checklist individually or with the help of a nurse available on request. At the end of the study visit, the investigator retrospectively recorded data on exacerbations over the previous 12 months and additional clinical and demographical information about the patient from the medical records.

The study was approved by the Ethics Committee of University Hospital Olomouc, Reference number 80/25. All patients gave informed consent for completing and processing the structured checklist and using their medical records data for this retrospective study.

### The structured checklist

**Figure 1:**
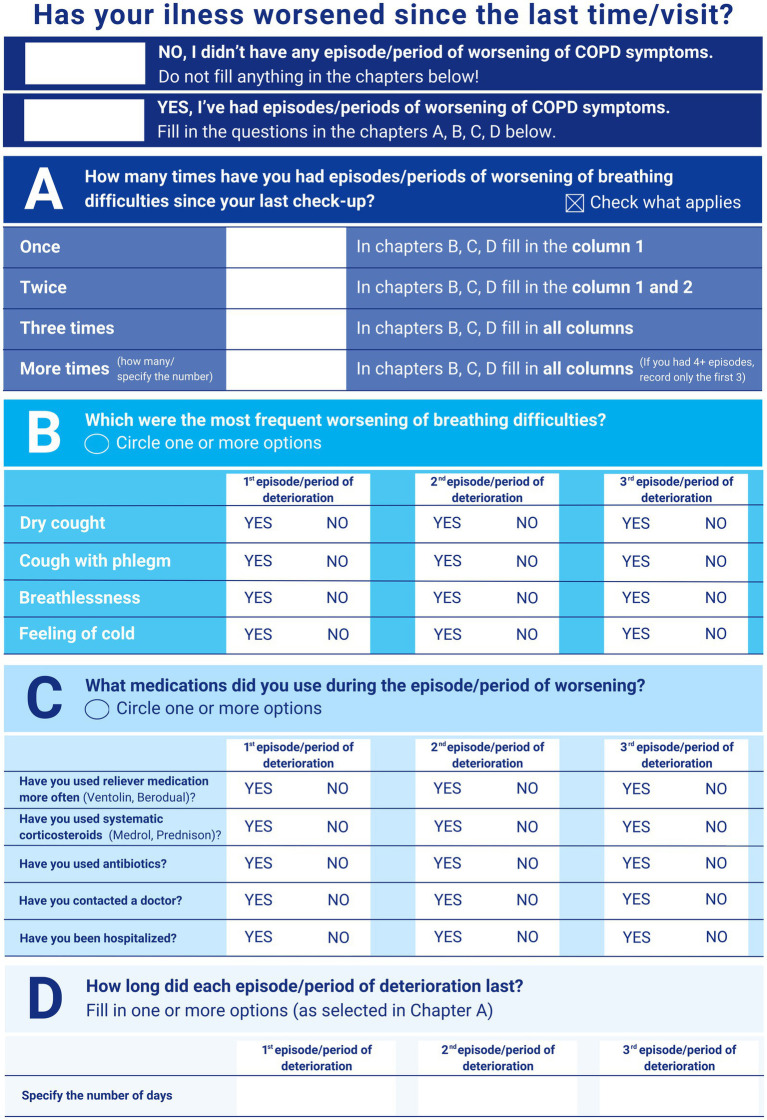
Structured checklist for the detection of past COPD exacerbations (English translation of the second page completed by patients).

The structured checklist was designed by expert panel, including the authors of this article, and based on our clinical experience, experience with the established scales and questionnaires, GOLD guidelines and practical considerations of the routine pulmonology practice (1, 2, 4). While the original two-page structured checklist is in Czech, Figure 1 gives the English translation of second page used by patients. The first page provides layperson-friendly information about the concept of exacerbation. This is not included in this paper. The second page, filled by the patient, collects data on past events. The structured checklist on the second page asks about the number of exacerbations since the last visit (Part A) and the symptoms selected by the patient (Part B). Part C covers event management, including pharmacotherapy and contact with a physician. Part D asks about the duration of past event(s) in days.

Based on the information provided by the patient, whether from a structured checklist or an interview, the reported exacerbations can be categorized according to the treatment used (1).

Mild exacerbations were treated with short-acting bronchodilators only, moderate events were managed with antibiotics and/or systemic corticosteroids, and severe exacerbations required hospitalization.

### Study outcomes

The main aim of our study was to test and evaluate the proposed structured checklist and its ability to detect past exacerbations, suitable for routine clinical practice. We compared the number of exacerbations (all and grouped by severity) that the patient had experienced since the last visit and that the patient reported in the structured checklist with the number of exacerbations obtained using usual conventional methods, i.e., a doctor-patient interview. The time since the last visit was also determined. Furthermore, we collected data from the medical records that were extracted for the last 12 months. Then we recalculated and converted the number of exacerbations in 12 months for each individual patient to the same period that had elapsed since their last visit. Based on the data provided by the patient in the structured checklist or interview, each exacerbation was classified by severity as mild, moderate, or severe.

To evaluate the structured checklist’s effectiveness, we compared the number of exacerbations since the last visit detected by the structured checklist with the number of exacerbations detected by interview. Furthermore, we compared the number of exacerbations since last visit detected by the structured checklist with the number of exacerbations recorded in medical records and converted to the period corresponding to the period since the last visit.

### Statistical analysis

Descriptive data are reported as means ± standard deviations (SD) or percentages, as appropriate. The incidence of exacerbations was summarized as per-year and per-person rate. Data from the entire cohort and from subgroups defined by the number of exacerbations reported in the structured checklist were analyzed. Differences between paired data within one group were tested using paired t-test, e.g., differences between the structured checklist, the interview or between medical records for each patient. Differences in continuous parameters between the two groups were tested by a two-sample *t*-test or Mann–Whitney U test, depending on the normality of the data. All hypotheses were tested on a 5% level of significance. The analyses were performed using Microsoft Excel and IBM SPSS Statistics, version 28.

## Results

A total of 350 COPD patients were enrolled. The characteristics of the patient cohort are shown in Table 1. 61.4% were male, and the mean age of the patients was 56.5 ± 14.8 years. Most patients had COPD GOLD stage 2 (64.3%) and stage 3 (28.6%). Mean FEV_1_ was 56.5 ± 14.8% of the predicted value. Among the comorbidities, hypertension (86.3%), hypercholesterolemia (48.0%), diabetes mellitus (29.4%), and atrial fibrillation (12.3%) were the most frequent. According to the medical records, patients had 494 exacerbations in the last year, with an average of 1.41 ± 1.6 per patient.

**Table 1:** Baseline characteristics of the analyzed patients’ population.

Characteristic	Analyzed population (*N* = 350)
Mean age (SD), years	56.5 (14.8)
Male patients, *n* (%)	215 (61.4)
Severity according to GOLD stages, *n* (%)	
Stage 1	8 (2.3)
Stage 2	225 (64.3)
Stage 3	100 (28.6)
Stage 4	17 (4.9)
Percentage of FEV_1_ predicted (mean, SD)	56.5 (14.8)
Number of exacerbations in the previous 12 months	
All exacerbations	494
Mild	120
Moderate	305
Severe	69
Mean number of exacerbations per patient in the previous 12 months (SD)	
All exacerbations	1.41 (1.6)
Mild	0.34 (0.9)
Moderate	0.87 (1.2)
Severe	0.20 (0.6)
Phenotypes according to Czech guidelines,^2^ *n* (%)	
Bronchitic phenotype	194 (55.4)
COPD and asthma overlap	105 (30.0)
Emphysematous phenotype	76 (21.7)
Frequent exacerbator phenotype	33 (9.4)
COPD and bronchiectasis overlap	23 (6.6)
Pulmonary cachexia phenotype	5 (1.4)
Comorbidities, *n* (%)	
Hypertension	302 (86.3)
Hypercholesterolemia	168 (48.0)
Diabetes mellitus	103 (29.4)
Atrial fibrillation	43 (12.3)
Ischemic heart disease	33 (9.4)
Peripheral vascular disease	33 (9.4)
History of myocardial infarction	32 (9.1)
History of stroke	12 (3.4)
History of transient cerebral ischemia	6 (1.7)
COPD treatment during the last 12 months, *n* (%)	
€Treatment containing ICS	227 (64.9)
Fixed combination of LABA+LAMA+ICS	111 (31.7)
Fixed combination of LABA+LAMA	99 (28.3)
Fixed combination of LABA+ICS	94 (26.9)
Inhaler containing LAMA	50 (14.3)
Inhaler containing LABA	33 (9.4)
Inhaler containing ICS	32 (9.1)
Free combination of LABA+LAMA+ICS	20 (5.7)

COPD, chronic obstructive pulmonary disease; FEV_1_, forced expiratory volume in 1 s; GOLD, Global Initiative for Chronic Obstructive Lung Disease; ICS, inhaled corticosteroid; LABA, long-acting beta_2_-agonist; LAMA, long-acting muscarinic antagonist; SD, standard deviation.

All patients completed the structured checklist without difficulties in approximately 1–2 min. Patients went through the form independently or asked the nurse for an explanation.

### Concordance between the structured checklist and the interview

**Figure 2:**
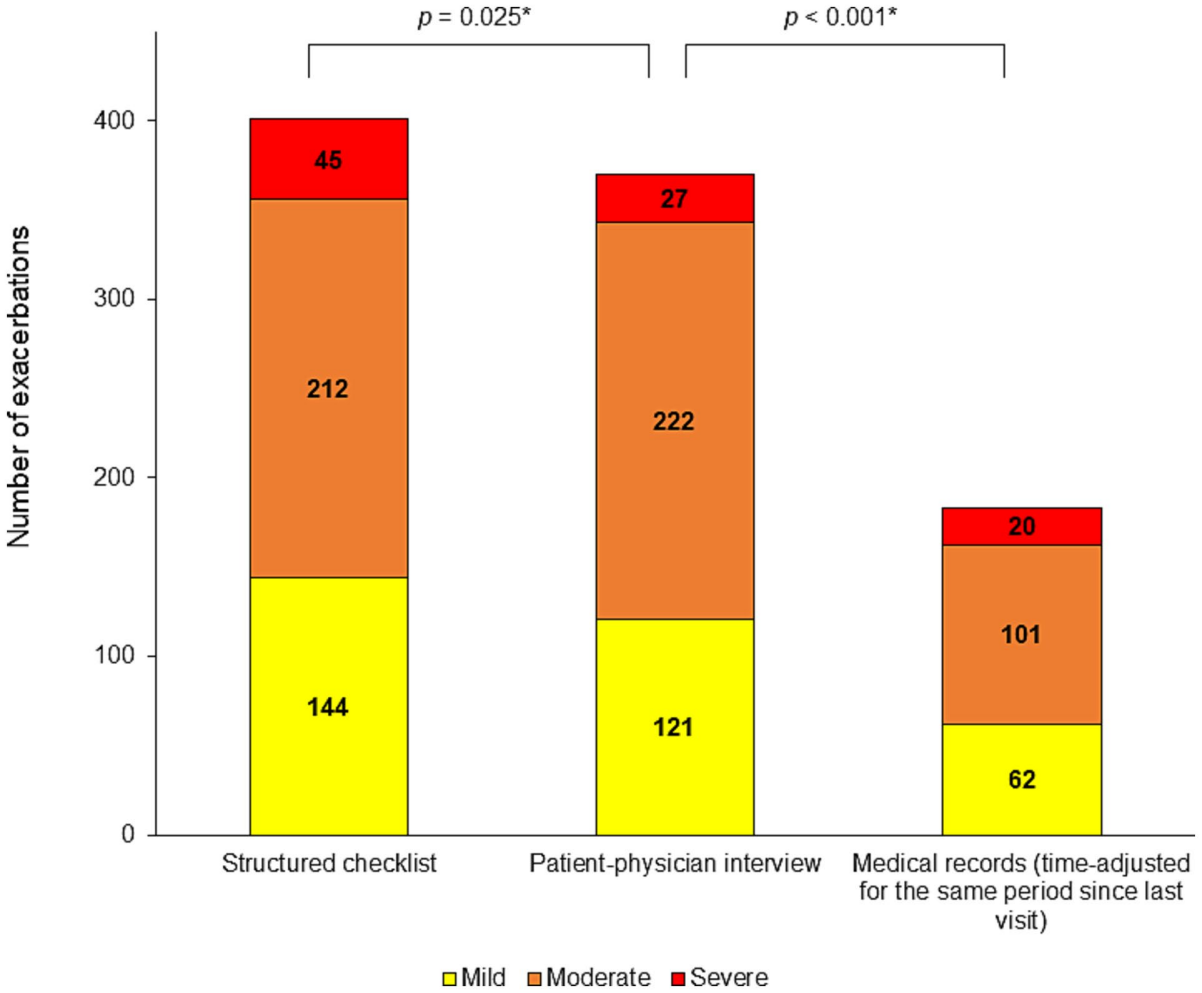
Number of detected exacerbations. Mild, moderate and, severe exacerbations since the last visit detected by the structured checklist, by the patient-physician interview and in the medical records (time-adjusted to cover the same period since the last visit as the other methods). *p-value for the comparison of all exacerbations number.

**Figure 3:**
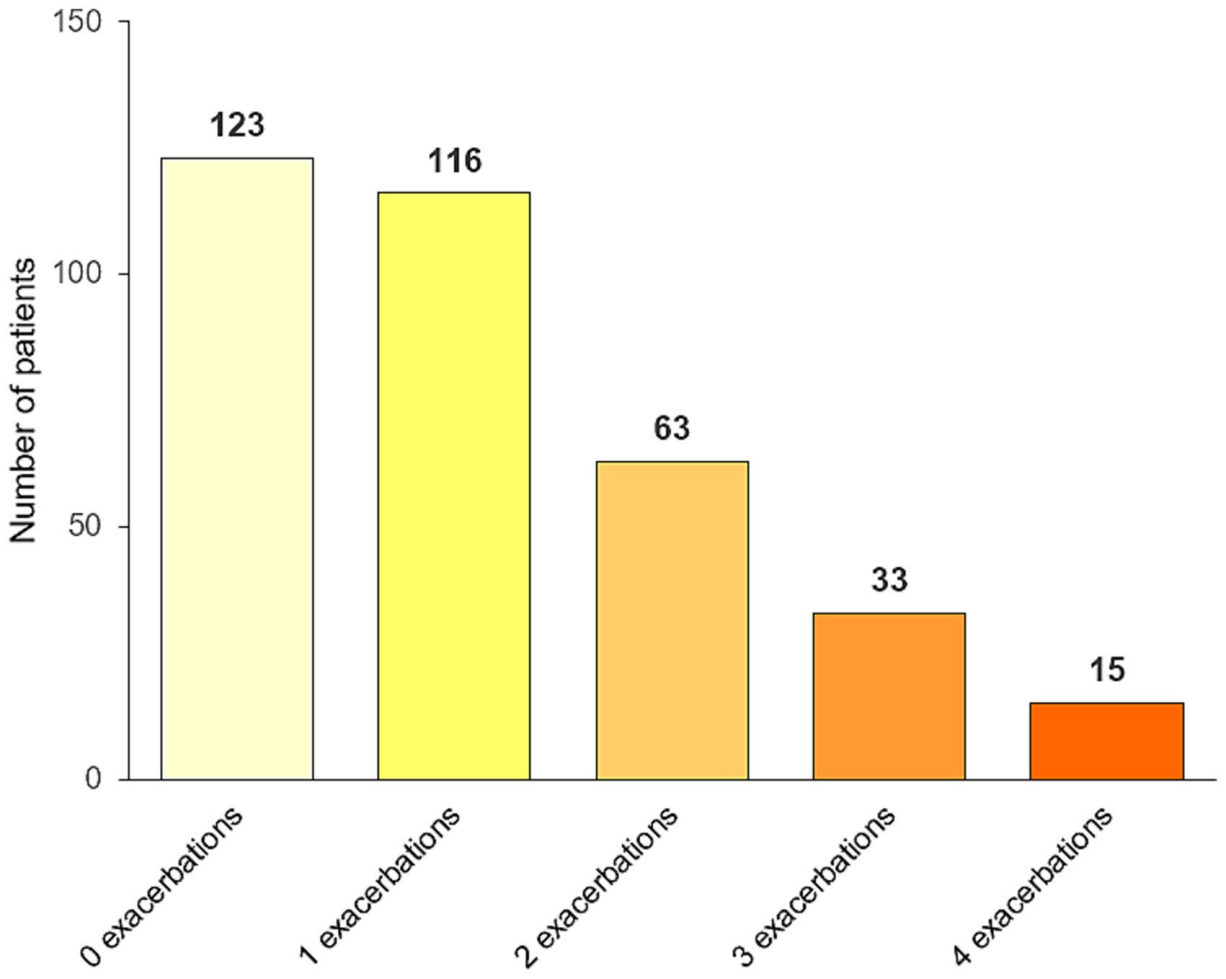
Proportion of patients with none, one, two, three, and four or more exacerbations since the last visit detected by the structured checklist.

The number of events detected by the structured checklist is shown in Table 2 and Figure 2. Supplementary Table S1 shows the exacerbation rates per patient. In the entire population of 350 patients, a total of 401 exacerbations since the last visit were detected by the structured checklist, while 370 exacerbations were detected by the interviews. The difference was statistically significant (*p* = 0.025). Using the structured checklist, approximately one third of patients (123; 35.1%) reported no exacerbation since their last visit, one third (116; 33.1%) experienced one exacerbation, and the last third reported two or more events (111; 31.7%) (Figure 3).

**Table 2:** Comparison of effectiveness in the detection of past exacerbations between the structured checklist and the patient-physician interview.

Structured checklist						Interview					*p-value*			
Parameter	Pts (*N*)	All events (*n*)	Mild (*n*)	Mod (*n*)	Sev (*n*)	All events (*n*)	Mild (*n*)	Mod (*n*)	Sev (*n*)	Number of patients reporded no event in interview (*N*)	All events	Mild	Mod	Sev
All patients	350	401	144	212	45	370	121	222	27	124	**0.025**	0.113	0.418	**0.003**
Subgroups by number of reported exacerbations in the structured checklist														
No events	123	0	0	0	0	14	1	11	2	112	**0.001**	0.319	**0.007**	0.158
1 event	116	116	53	56	7	110	34	71	5	10	0.109	**<0.001**	**<0.001**	0.158
2 events	63	126	44	69	13	104	36	59	9	2	**<0.001**	0.261	0.105	0.159
3 events	33	99	21	63	15	87	18	63	6	0	**0.008**	0.62	1	**0.027**
4 events	15	60	26	24	10	55	32	18	5	0	0.642	0.567	0.32	0.055

Numbers of exacerbation since last visit assessed by structured checklist and by physician-patients interview.

Mod, moderate; *N*, number of patients; *n*, number of events; Pts, patients; Sev, severe. Bolding indicates significant data. The severity of exacerbations according to GOLD was determined based on the treatment of the exacerbation that the patient reported in the structured checklist or that the physician identified during the interview.

In the overall study population, the structured checklist captured numerically more mild exacerbations (144 vs. 121; *p* = 0.113) and significantly more severe exacerbations (45 vs. 27; *p* = 0.003) compared to the patient-physician interview. In subgroups defined by the number of exacerbations reported in the structured checklist, significantly higher numbers of all exacerbations were detected by the structured checklist compared to the interview in patients who reported two or three exacerbations (126 vs. 104, *p* < 0.0001; 99 vs. 87, *p* = 0.008, respectively). In patients with one exacerbation since their last visit, the structured checklist identified only a numerically higher number of all exacerbations (116 vs. 110; *p* = 0.109) but a significantly higher number of mild exacerbations (53 vs. 34; *p* = 0.0001) and a significantly lower number of moderate exacerbations (56 vs. 71; *p* = 0.0009) compared with the patient-physician interview.

Of the 53 patients who reported only 1 mild exacerbation on the structured checklist, there were 10 patients in whom the interview did not detect any exacerbation, in another 31 patients the physician interview detected one mild exacerbation consistent with the structured checklist, and in 12 patients the physician interview rated the exacerbations as moderate. In addition, the interview then rated 3 other patients as having mild exacerbations, although the structured checklist rated these exacerbations differently as mild (Table 2).

In the group of 123 patients who reported no exacerbation in the structured checklist, the interview with the physician also did not detect any exacerbation in 112 patients, while in 11 patients (8.9%), the interview detected some exacerbations, including one mild, 11 moderate, and two severe events. The difference between structured checklist and the interview was significant for all and moderate exacerbations (*p* = 0.001 and 0.007, respectively) (Table 2).

Among the 116 patients who reported one exacerbation in the structured checklist, there were 10 patients (8.6%) in whom the interview did not detect any event and four patients in whom the interview detected two exacerbations. In addition, the patient-physician interview did not detect any exacerbation in two patients who reported two exacerbations in the structured checklist (Table 2).

### Concordance between the structured checklist and the medical records

The mean time since the last visit to the physician was 149 days (5 months) in the overall study population. Table 3 shows the number of exacerbations identified in the medical chart review of the last 12 months, adjusted to cover the same period since the last visit, as assessed by the structured checklist and the interview. The exacerbation rates per patient are shown in Supplementary Table S2, and the number of exacerbations from which the time-adjusted numbers and rates were calculated are shown in Supplementary Table S3.

**Table 3:** Comparison of effectiveness in the detection of past exacerbations between the structured checklist and medical records.

Structured checklist						Mean time since last visit, in days (SD)	Medical records (time-adjusted)				*p-value*			
Parameter	Pts (*N*)	All events (*n*)	Mild (*n*)	Mod (*n*)	Sev (*n*)		All events (*n*)	Mild (*n*)	Mod (*n*)	Sev (*n*)	All events	Mild	Mod	Sev
All patients	350	401	144	212	45	149 (107)	183	62	101	20	**<0.001**	**<0.001**	**<0.001**	**<0.001**
Subgroups by number of reported exacerbations in the structured checklist														
No event	123	0	0	0	0	146 (122)	21	5	13	3	**<0.001**	**0.004**	**<0.001**	**0.0026**
1 event	116	116	53	56	7	154 (102)	45	17	25	3	**<0.001**	**<0.001**	**<0.001**	0.1
2 events	63	126	44	69	13	156 (102)	50	17	27	6	**<0.001**	**<0.001**	**<0.001**	**0.023**
3 events	33	99	21	63	15	142 (89)	44	12	27	4	**<0.001**	0.121	**<0.001**	**0.021**
4 events	15	60	26	24	10	115 (53)	23	11	9	3	**<0.001**	**0.046**	**0.002**	**0.014**

Mod, moderate; *N*, number of patients; *n*, number of events; Pts, patients; Sev, severe; SD, standard deviation. Bolding indicates significant data. The severity of exacerbations according to GOLD was determined based on the treatment of the exacerbation that the patient reported in the structured checklist or that was listed in the medical records.

Number of exacerbations since the last visit according to the structured checklist, time elapsed since the last visit, and number of exacerbations obtained from medical records and recalculated to the same period corresponding to the time since the last visit.

The structured checklist, asking about the period since the last visit, detected more exacerbations than the review of medical records, recalculated over the same period. The structured checklist identified more than twice as many events in the overall study population and in patients that reported one or more events. The differences reached statistical significance (Table 3; Figure 2).

In the subgroup of 123 patients who reported no exacerbation in the structured checklist, there were 78 patients who had no exacerbation in the past year also in their medical records. Other 45 of these patients had a record of at least one exacerbation in the last year; the time-adjusted number of events in these patients was 21 since last visit, and moderate intensity predominated. In 10 of these patients, exacerbations were also noted during the interview.

### Symptoms and duration of exacerbations

The presence of symptoms (cough, shortness of breath, and feeling cold) and the duration of each exacerbation that the patient experienced since the last visit and reported on the structured checklist are shown in Table 4. The most prevalent symptoms were dyspnea (81.3%) and productive cough (61.1%). Feeling cold and dry cough were present in 44.1 and 32.2% of events, respectively. Two symptoms were present simultaneously in 53.9% of events. The mean duration of exacerbation was 9.7 days in the overall population.

**Table 4:** Symptoms and duration of exacerbations since last visit, reported using the structured checklist.

Parameter	Exacerbations (*n*)	Dyspnea (*n*, %)	Productive cough (*n*, %)	Feeling cold (*n*, %)	Dry cough (*n*, %)	Mean duration in days (SD)
All patients	401	326 (81.3)	249 (62.1)	177 (44.1)	129 (32.2)	9.7 (7.3)
Subgroups by number of reported exacerbations in the structured checklist						
1 event	116	92 (79.3)	63 (54.3)	48 (41.4)	47 (40.5)	10.9 (9.8)
2 events	126	105 (83.3)	80 (63.5)	60 (47.6)	42 (33.3)	10.0 (6.8)
3 events	99	86 (86.9)	76 (76.8)	56 (56.6)	29 (29.3)	9.0 (4.4)
4 events	60	43 (71.7)	30 (50.0)	13 (21.7)	11 (18.3)	7.8 (6.0)

*n*, number of events; SD, standard deviation.

A total of 139 of 225 patients with moderate COPD (61.8%), 70 of 100 patients with severe COPD (70.0%), and 10 of 17 patients with very severe COPD (58.8%) experienced at least one exacerbation since the previous visit, with a mean number of detected exacerbations of 1.07, 1.26 and 1.24 events per patient, respectively (Table 5).

**Table 5:** Exacerbation rates since the last visit according to COPD stages detected by the structured checklist and during first and second year before the study visit in the medical records.

COPD stage	Pts (*N*)	Structured checklist		First year (24 to 12 months) before the study visit		Second year (last 12 months) before the study visit	
		Pts with ≥ 1 event (%)	Event rate per patient	Pts with ≥ 1 event (%)	Event rate per patient and year	Pts with ≥ 1 event (%)	Event rate per patient and year
Mild	8	8 (100)	1.75	5 (62.5)	1	5 (62.5)	1.13
Moderate	225	139 (61.8)	1.07	133 (59.1)	1.08	147 (65.3)	1.32
Severe	100	70 (70.0)	1.26	54 (54.0)	0.81	73 (73.0)	1.30
Very severe	17	10 (58.8)	1.24	15 (88.2)	2.31	16 (94.1)	3.35
All stages	350	227 (64.9)	1.15	207 (59.1)	1.05	241 (68.9)	1.41

COPD, chronic obstructive pulmonary disease; *N*, number of patients; Pts, patients.

## Discussion

We have tested and evaluated a structured checklist for detection of COPD exacerbation history that is suitable for routine clinical practice, and tested its effectiveness in outpatient settings in the cohort of 350 patients from 35 centers across the Czech Republic. Study participants mostly had moderate and severe COPD. Completing the structured checklist did not significantly burden the patients, as it took approximately 1–2 min, comparable to other established tools, such as the CAT questionnaire.

Compared with the standard method of screening exacerbation history, i.e., the patient-physician interview, our structured checklist detected 8.4% more overall exacerbations (*p* = 0.025). We also focused on mild exacerbations, that may not require a visit to a physician and may remain unreported to healthcare professionals. Although not statistically significant, the structured checklist detected 19.0% more mild exacerbations than the interview (*p* = 0.113) in the overall study population.

Using the structured checklist, a higher number of exacerbations was found compared to the patient-physician interview, regardless of how many exacerbations the patient had experienced since the previous visit. However, the structured checklist seemed even more sensitive than the interview in those patients who reported two or three exacerbations since the last visit (*p* = 0.00002; *p* = 0.008, respectively) and specifically in those who reported only one mild exacerbation (*p* = 0.0001). The interview detected no event in 18.9% of patients who declared one mild exacerbation in the structured checklist. For these patients, the structured checklist was more sensitive than the interview. On the contrary, in 8.9% of all patients who declared no event in the structured checklist, the exacerbation was identified during the interview with the physician. In these patients, the structured checklist result was considered a false negative.

The patient-physician interview and structured checklist completion occurred during one study visit, so it can be assumed that the physicians focused more carefully on detecting exacerbations during the interviews than usual. Therefore, we also compared the number of exacerbations detected using the structured checklist with those recorded in the patient’s medical charts in the last 12 months, which our study procedure could not have influenced. The numbers of exacerbations obtained from medical records were adjusted to cover the same time period as the structured checklist and interview. Our structured checklist identified more than twice as many events in overall study population and in most subgroups by number of reported exacerbations, compared to data from medical records, which were recalculated for the same period corresponding to the time since the last visit for each patient.

The incidence of exacerbations in patients with COPD varies across studies. Data from an extensive UK database including 340,515 COPD patients published in 2022 show that in the previous year, 53.2% of patients experienced no exacerbations, 20.0% experienced one moderate exacerbation, 9.4% experienced two moderate exacerbations, 9.9% experienced three or more moderate exacerbations, 5.6% experienced one severe exacerbation, 1.2% experienced two severe exacerbations and 0.7% experienced three or more severe exacerbations (5). Another study published in 2021 that examined the effect of exacerbation history on subsequent exacerbations using the data from a large population of 250,723 COPD patients summarized that 78% of patients had no prior exacerbation in the previous year, 11% had one moderate and 11% had severe or multiple exacerbations at the baseline (21). Data from the ECLIPSE study (2010) involving 2,138 patients showed that 53% of patients had no exacerbation in the previous year, 47% had at least one exacerbation in the previous year, and 29% had at least two exacerbations. In this study, 39, 52, and 62% of patients with COPD stages 2, 3, and 4, respectively, experienced at least one exacerbation (7). In our study, 61.8, 70, and 58.8% of patients with moderate, severe, and very severe COPD, respectively, had experienced at least one exacerbation since the previous visit. The number of exacerbations detected by our structured checklist was higher than the number of exacerbations per year in the cited studies (5, 7, 21). It could be due to the larger representation of patients with more severe stages of COPD and the fact that the structured checklist was completed from May to June 2023, covering approximately five previous months (time since the last visit), during which higher incidence of respiratory infections is common and increases the frequency of exacerbations (22).

Mild exacerbations accounted for 73 to 74% of all exacerbations in the FLAME and SPARK studies (17, 18), and approximately 38% of all events in the RESTORE study (10). In our study, mild exacerbations detected by the questionnaire accounted for 35.9% of all exacerbations, similar to the RESTORE study and significantly less than in the SPARK and FLAME studies. Using our structured checklist, we found 19% more mild exacerbations than in the patient-physician interview and 2.3 times more than in the review of medical records. Compared to SPARK and FLAME studies, the incidence of mild exacerbations remained underestimated even using our structured checklist. It seems that it is still difficult to establish an accurate history of COPD exacerbations at present (23), and the result of our study shows that using a simple structured checklist could improve the detection of past exacerbations.

Our study has several limitations. First, the structured checklist we tested was not developed according to the precise and strict criteria required for creating a questionnaire. This is related to some of its characteristics. To keep the structured checklist as simple and practical as possible, we decided to leave out several details about the exacerbation, such as sputum purulence, emergency room visits, or duration of systemic corticosteroids or antibiotic treatment. Consequently, the results of the structured checklist cannot be used for the Anthonisen classification of exacerbations (24), and the physician gets rather superficial knowledge about the treatment of the reported event, although this will allow the severity of the exacerbation to be determined according to GOLD. These limitations can be improved by future modifications of the structured checklist, eg, by adding new questions. In addition, the structured checklist does not allow for collecting details about the fourth and subsequent events. Nevertheless, it still alerts the physician to the higher number of exacerbations and gives the opportunity to ask the patient for more information.

Another important limitation is that the effectiveness of detecting exacerbations using the structured checklist in our study was compared with the doctor-patient interview, as a usual method in clinical practice, while no standard method for detecting exacerbations used in clinical trials (e.g., diaries or electronic diaries) was used for comparison. Bias may be introduced by the timing of the structured checklist’s completion and the interview with the physician. Both procedures took place at the same visit, so they may have influenced each other. It is also likely that the physician was more intently and carefully focused on detecting exacerbations during the interview than he or she would have done in everyday clinical practice.

The final limitation we would like to mention is that the structured checklist was created and tested by respiratory doctors in the Czech Republic, among Czech patients, and in the Czech language. Its transferability to other languages and countries has not yet been tested.

## Conclusion

We have tested a structured checklist that is easy to use in routine clinical practice to detect past COPD exacerbations. Using standard patient-physician interview as a reference tool to screen exacerbation history, we demonstrated that our structured checklist significantly improved the detection of past exacerbations, including mild events. Compared to the records in the medical charts, which reflect the detection of exacerbations in routine clinical practice, our structured checklist detected more than twice as many exacerbations. The structured checklist allows the determination of severity and other clinical characteristics of exacerbations and also records the treatment.

While it is still difficult to establish an accurate history of COPD exacerbations, our work suggests that using a simple structured checklist for detection of exacerbation history like ours could reduce the number of undetected events and improve the management of COPD. At the same time, our work shows some requirements for future research. In the future, it will be necessary to develop a questionnaire based on strict and precise criteria. It will also be necessary to test the questionnaire by comparing it with standard methods for detecting exacerbations used in clinical trials (e.g., diaries or e-diaries).

## Data Availability

The data supporting this manuscript may be shared upon reasonable request to the corresponding author.
