# Supplementary material for: New instrument for effective detection of a history of COPD exacerbations, including usually unreported events
Source: Front Med (Lausanne). 2025 Sep 19;12:1630338. doi: 10.3389/fmed.2025.1630338 (PMC12490996; doi:10.3389/fmed.2025.1630338)
Supplement: Supplementary file 1 [file Table_1.docx]

**Supplementary material**

Zatloukal J, Volakova E, Kovacikova J, et al. New instrument for effective detection of a history of COPD exacerbations, including usually unreported events.

List of supplementary tables

**Table S1:** Comparison of effectiveness in the detection of past exacerbations between the structured checklist and patient-physician interview (event rate per patient)

**Table S2:** Comparison of effectiveness in the detection of past exacerbations between the structured checklist and medical records (adjusted for the same time since the last visit as the structured checklist; event rate per patient)

**Table S3:** Number of exacerbations in the last 12 months detected in the medical records (data for the calculation of time-adjusted values shown in Table 3 in the article)

**Table S1:** Comparison of effectiveness in the detection of past exacerbations between the structured checklist and patient-physician interview (event rate per patient)

| **Structured checklist** | | | | | | **Interview** | | | |
| --- | --- | --- | --- | --- | --- | --- | --- | --- | --- |
|  | Pts  (N) | All events | Mild | Mod | Sev | All events | Mild | Mod | Sev |
|  |  | Event rate (number per patient) | | | | Event rate (number per patient) | | | |
| All patients | 350 | 1.15 | 0.41 | 0.61 | 0.13 | 1.06 | 0.35 | 0.63 | 0.08 |
| **Subgroups by number of reported exacerbations in the structured checklist** | | | | | | | | | |
| No events | 123 | 0.00 | 0.00 | 0.00 | 0.00 | 0.11 | 0.01 | 0.09 | 0.02 |
| 1 event | 116 | 1.00 | 0.46 | 0.48 | 0.06 | 0.95 | 0.29 | 0.61 | 0.04 |
| 2 events | 63 | 2.00 | 0.70 | 1.10 | 0.21 | 1.65 | 0.57 | 0.94 | 0.14 |
| 3 events | 33 | 3.00 | 0.64 | 1.91 | 0.45 | 2.64 | 0.55 | 1.91 | 0.18 |
| 4 events | 15 | 4.00 | 1.73 | 1.60 | 0.67 | 3.67 | 2.13 | 1.20 | 0.33 |

**Abbreviations:** Mod, moderate; N, number of patients; no., number; Pts, patients; Sev, severe, N, number of patients; n, number of events

**Table S2:** Comparison of effectiveness in the detection of past exacerbations between the structured checklist and medical records (adjusted for the same time since the last visit as the structured checklist; event rate per patient)

| **Structured checklist** | | | | | | **Medical records  (time-adjusted)** | | | |
| --- | --- | --- | --- | --- | --- | --- | --- | --- | --- |
|  | Pts (N) | All events | Mild | Mod | Sev | All events | Mild | Mod | Sev |
|  |  | Event rate (number per patient) | | | | Event rate (number per patient) | | | |
| All patients | 350 | 1.15 | 0.41 | 0.61 | 0.13 | 0.52 | 0.18 | 0.29 | 0.06 |
| **Subgroups by number of reported exacerbations in the structured checklist** | | | | | | | | | |
| No event | 123 | 0.00 | 0.00 | 0.00 | 0.00 | 0.17 | 0.04 | 0.10 | 0.03 |
| 1 event | 116 | 1.00 | 0.46 | 0.48 | 0.06 | 0.39 | 0.15 | 0.21 | 0.03 |
| 2 events | 63 | 2.00 | 0.70 | 1.10 | 0.21 | 0.80 | 0.27 | 0.43 | 0.10 |
| 3 events | 33 | 3.00 | 0.64 | 1.91 | 0.45 | 1.32 | 0.36 | 0.82 | 0.14 |
| 4 events | 15 | 4.00 | 1.73 | 1.60 | 0.67 | 1.55 | 0.72 | 0.62 | 0.21 |

**Abbreviations:** Mod, moderate; N, number of patients; no., number; Pts, patients; Sev, severe, N, number of patients; n, number of events

**Table S3:** Number of exacerbations in the last 12 months detected in the medical records in all patients and in subgroups by number of reported exacerbations in the structured checklist (data for the calculation of time-adjusted values shown in Table 3 in the article)

|  |  | **Exacerbations detected in the medical records (last 12 months)** | | | | | | | |
| --- | --- | --- | --- | --- | --- | --- | --- | --- | --- |
|  |  | Number of events (n) | | | | Event rate (number per patient) | | | |
|  | Pts (N) | All events | Mild | Mod | Sev | All events | Mild | Mod | Sev |
| All patients | 350 | 494 | 120 | 305 | 69 | 1.41 | 0.34 | 0.87 | 0.20 |
| **Subgroups by number of reported exacerbations in the structured checklist** | | | | | | | | | |
| No event | 123 | 71 | 13 | 43 | 15 | 0.58 | 0.11 | 0.35 | 0.12 |
| 1 event | 116 | 117 | 31 | 77 | 9 | 1.01 | 0.27 | 0.66 | 0.08 |
| 2 events | 63 | 118 | 28 | 71 | 19 | 1.87 | 0.44 | 1.13 | 0.30 |
| 3 events | 33 | 117 | 21 | 83 | 13 | 3.55 | 0.64 | 2.52 | 0.39 |
| 4 events | 15 | 71 | 27 | 31 | 13 | 4.73 | 1.80 | 2.07 | 0.87 |

**Abbreviations:** Mod, moderate; no., number; Pts, patients; Sev, severe, N, number of patients; n, number of events
